# Preferential transduction of parvalbumin-expressing cortical neurons by AAV-mDLX5/6 vectors

**DOI:** 10.3389/fnins.2023.1269025

**Published:** 2024-02-12

**Authors:** Padideh Yazdan-Shahmorad, Shane Gibson, Joanne C. Lee, Gregory D. Horwitz

**Affiliations:** ^1^Department of Electrical and Computer Engineering, University of Washington, Seattle, WA, United States; ^2^Washington National Primate Research Center, Seattle, WA, United States; ^3^Department of Physiology and Biophysics, University of Washington, Seattle, WA, United States

**Keywords:** AAV, DLX5/6, enhancer, macaque, cell type-specificity, parvalbumin, calretinin

## Abstract

A major goal of modern neuroscience is to understand the functions of the varied neuronal types that comprise the mammalian brain. Toward this end, some types of neurons can be targeted and manipulated with enhancer-bearing AAV vectors. These vectors hold great promise to advance basic and translational neuroscience, but to realize this potential, their selectivity must be characterized. In this study, we investigated the selectivity of AAV vectors carrying an enhancer of the murine Dlx5 and Dlx6 genes. Vectors were injected into the visual cortex of two macaque monkeys, the frontal cortex of two others, and the somatosensory/motor cortex of three rats. Post-mortem immunostaining revealed that parvalbumin-expressing neurons were transduced efficiently in all cases but calretinin-expressing neurons were not. We speculate that this specificity is a consequence of differential activity of this DLX5/6 enhancer in adult neurons of different developmental lineages.

## Introduction

The mammalian brain consists of many distinct neuronal types. How each type contributes to circuit function and behavior is poorly understood. To fill this gap in knowledge, techniques are needed to record and modulate the activity of individual types. Genetically encoded reporters and actuators of neural activity have been used fruitfully in this endeavor for many years in transgenic animals. These days, similar manipulations can be made in wild-type animals via viral vector-mediated gene delivery. Effective use of these tools requires knowledge of which neuronal types each vector transduces and which they do not.

In this report, we describe transgene expression patterns produced by intracerebral injections of AAVs containing an enhancer of the mouse distal-less 5 and 6 (*Dlx5/6*) genes (Zerucha et al., 2000; Dimidschstein et al., 2016). In transgenic mice, this enhancer drives transgene expression in many, and possibly all, telencephalic inhibitory interneuronal types (Stühmer et al., 2002). Packaged in AAV, it drives transgene expression in GABAergic neurons, but how efficiently these AAV vectors transduce different types of GABAergic neurons is incompletely understood.

Knowing the answer to this question is critical for the application of these vectors in experimental and clinical settings. For example, one application of AAV-DLX5/6 vectors is to suppress the activity of excitatory projection neurons via the excitation of local inhibitory neurons. Some inhibitory neurons target other inhibitory neurons, however, thereby disinhibiting projection neurons. Knowing whether AAV-DLX5/6 vectors preferentially transduce inhibitory neurons that target other inhibitory neurons is important for understanding the likelihood of this effect.

## Results

One class of GABAergic neuron in the cerebral cortex expresses the calcium binding protein, parvalbumin (PV). These neurons are particularly common in macaque V1, where they account for 50–74% of the total GABAergic population (Van Brederode et al., 1990; Kooijmans et al., 2020). In a previous study, we showed that an AAV-mDLX5/6 vector injected into macaque V1 transduced PV+ neurons with a selectivity of 86% (De et al., 2020).

**FIGURE 1:**
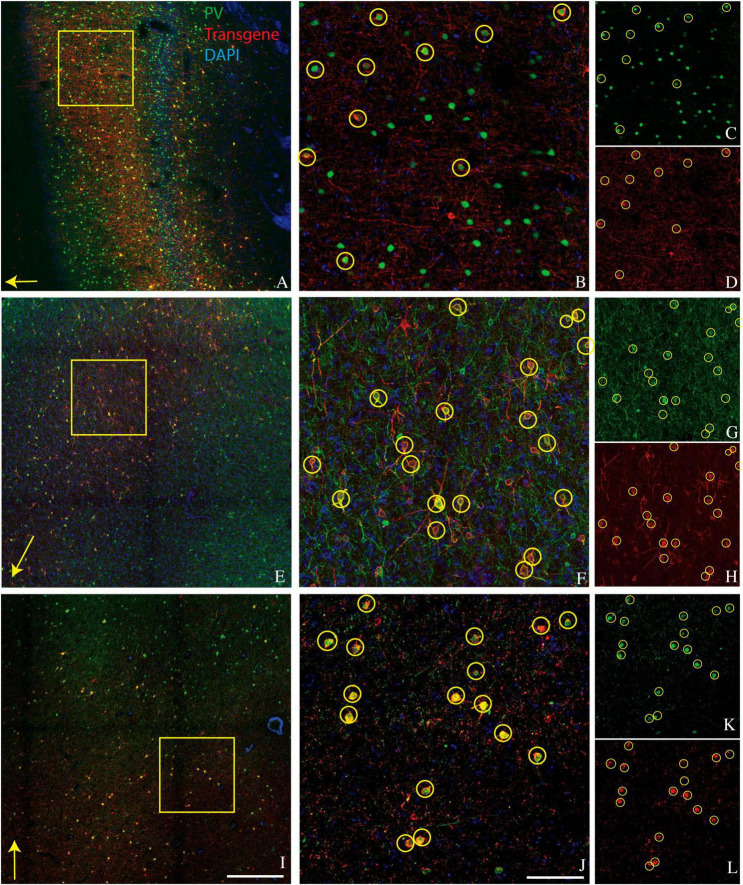
Representative images showing colocalization of parvalbumin (green) and transgene (red) in tissue sections from three monkeys. Sections are from monkey V **(A–D)**, monkey J **(E–H)**, and monkey M **(I–L)**. Transgenes are ChR2-mCherry for monkey V and ChRmine-mScarlet for monkeys J and M. **(A,E,I)** Images collected at 10x. Scale bar in **(I)** is 400 μm and applies to all 10x images. Arrows indicate the apical direction. Squares represent the area shown at 20x in **(B,F,J)**. Scale bar in **(J)** is 100 μm and applies to all 20x images. Circles indicate double-labeled cells. Panels **(C,D)**, **(G,H)**, and **(K,L)** are single-channel images of panels **(B,F,J)**, respectively. Tissue sections from monkey J **(E–H)** and monkey M **(I–L)** were triple-stained for parvalbumin, calretinin, and mScarlet and are identical to those in Figures 2E–H and 2I–L, respectively.

A goal of the current study was to determine whether this bias for PV+ neurons extended to other cortical areas. We injected AAV9-mDLX5/6-ChR2-mCherry into the extrastriate visual cortex of a second macaque (monkey V) and AAV(PHP.eB)-mDLX5/6-ChRmine-mScarlet into the frontal cortex of two others (monkeys J and M). Consistent with our earlier findings, PV+ neurons were transduced with high efficiency (Figure 1). The percentage of transduced neurons that was PV+ was high, albeit lower than in our previous study (50% in monkey V, 66% in monkey J, and 73% in monkey M; see Table 1 for cell counts). Near the injection site, the percentage of PV+ neurons that was transduced was lower and more variable (19% in monkey V, 29% in monkey M, and 53% in monkey J). This inter-animal variability is likely at least partially due to technical differences across experiments, an issue we return to in the Discussion.

**TABLE 1:** Details of virus injections and cell counts.

Animal ID	Sex	Date of birth	Date of injection	Date of sacrifice	Serotype and payload	Titer (vg/ml)	Injected Vol (μl)	Number of sections	Cell counts				
									**PV**	**CR**	**Red fluorescent protein (RFP)**	**PV spots colocalized with RFP**	**CR spots colocalized with RFP**
Monkey A	M	6/7/2005	12/18/2018	2/28/2020	AAV1, ChR2-mCherry	1 × 10^13^	30	1	– –	3664 –	742 –	– –	6 –
Monkey V	F	6/13/2006	8/24/2017	10/26/2017	AAV9, ChR2-mCherry	9 × 10^12^	10	1	2978 –	– –	1134 –	570 –	– –
Monkey J	M	9/01/1999	5/04/2023	6/08/2023	PHP.eB, ChRmine-mScarlet	7 × 10^12^	7	2	1257 961	515 –	896 883	596 572	1 –
Monkey M	M	4/25/2010	5/15/2023	6/26/2023	PHP.eB, ChRmine-mScarlet	7 × 10^12^	21	2	2908 4506	6146 –	1620 1362	1114 1059	31 –
Rat 1	F	3/14/2022	1/25/2023	2/22//2023	PHP.eB, ChrimsonR-tdTomato	4 × 10^12^	3	2	186 –	– 191	219 80	111 –	– 5
Rat 2	F	2/23/2023	9/11/2023	10/12/2023	*ChrimsonR-tdTomato	*	3	4	259 –	77 –	261 –	133 –	6 –
Rat 3	F	2/23/2023	9/13/2023	10/12/2023	PHP.eB, ChrimsonR-tdTomato	6 × 10^13^	3	3	119 –	40 –	71 –	32 –	4 –

*3 μl of AAV1 (6.1 × 10^13^ vector genomes/ml) and 3 μl of AAV (PHP.eB) (1.3 × 10^13^ vector genomes/ml) were injected into the left and right hemisphere, respectively.

A secondary goal was to determine how many of the remaining transduced cells expressed calretinin (CR). Cortical CR+ interneurons are similar to cortical PV+ interneurons in that both are GABAergic, relatively common, and consist of multiple morphologically and physiologically distinct subtypes (DeFelipe, 1997; Tremblay et al., 2016). However, CR+ and PV+ neurons are distinct populations with different developmental lineages, laminar distribution, and functions (Callaway, 2004; Fogarty et al., 2007; Ma et al., 2013; Kooijmans et al., 2020). Importantly, many cortical and hippocampal CR+ neurons preferentially target other inhibitory interneurons (Gulyas et al., 1996; Callaway, 2004).

**FIGURE 2:**
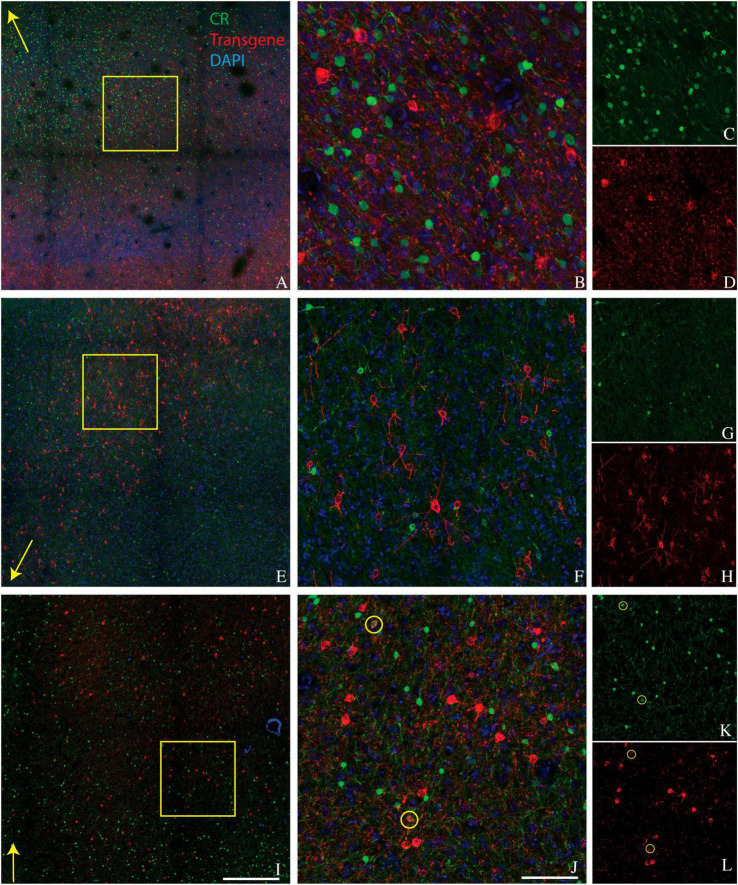
Colocalization of calretinin (green) and transgene (red) in tissue sections from monkey A **(A–D)**, monkey J **(E–H)**, and monkey M **(I–L)**. Transgenes are ChR2-mCherry for monkey A and ChRmine-mScarlet for monkeys J and M. Conventions are as in Figure 1.

We conducted CR staining on tissues from monkeys A, J, and M. We were unable to stain for CR in monkey V due to tissue degradation during storage. Very few transduced neurons were CR+ (6/742 in monkey A, 1/896 in monkey J, and 31/2982 in monkey M) (Figure 2). The proportion of CR+ neurons near the injection site that was transduced was also quite low (6/3664 in monkey A, 1/515 in monkey J, and 31/6146 in monkey M). We conclude that AAV-mDLX5/6 vectors are not pan-GABAergic but, instead, avoid CR+ neurons.

**FIGURE 3:**
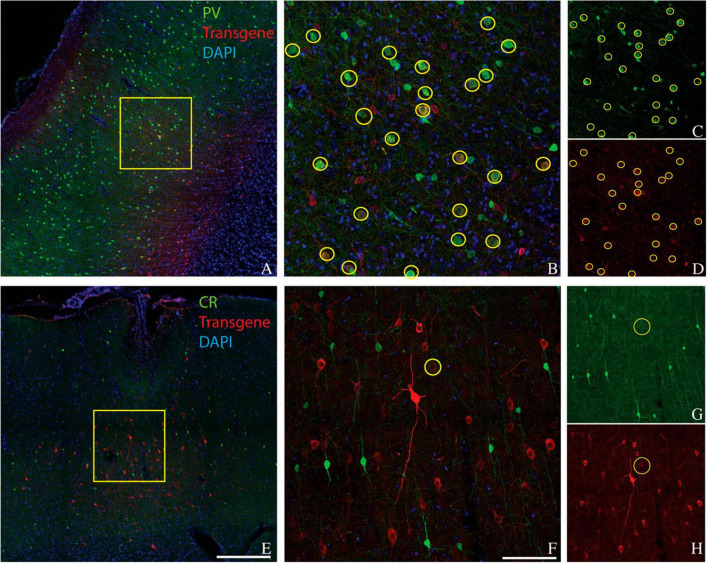
Colocalization of inhibitory neuron markers (green) and ChrimsonR-td Tomato (red) in the somatosensory/motor cortex of rat 1. Inhibitory markers are parvalbumin **(A–D)** and calretinin **(E–H)**. Scale bars are 400 μm **(A,E)** and 100 μm **(B,F)**. Conventions are as in Figure 1.

We considered the possibility that the bias of AAV-mDLX5/6 vectors for PV+ neurons over CR+ neurons was primate-specific. To test this idea, we injected AAV(PHP.eB)-mDLX5/6-ChrimsonR-tdTomato into the somatosensory/motor cortex of three rats (Figure 3). On average, 49% of transduced neurons were PV+ (range: 45–51%, see Table 1 for counts from individual animals). Similarly, the percentage of PV+ neurons near the injection site that was transduced was 46% (range: 27–60%). In contrast, only 3% (2–6%) of transduced neurons were CR+, and only 7% (3–10%) of the CR+ neurons near the injection site were transduced. These results show that the preferential transduction of PV+ neurons over CR+ neurons by AAV-mDLX5/6 vectors is not specific to primates.

We considered the possibility that the AAV capsids used in these experiments were responsible for the preference for PV+ neurons over CR+ neurons. A complete assessment of the role of AAV serotype is beyond the scope of this report, but as an initial step we injected AAV1-mDLX5/6-ChrimsonR-tdTomato into one rat, directly across the midline from the AAV(PHP.eB) injection site. Transduction patterns were similar across hemispheres, which is why data from both injections were pooled in the counts provided above (see Table 1 for breakdown by serotype).

## Discussion

We injected AAV-mDLX5/6 vectors into the visual and frontal cortices of rhesus monkeys and into the somatosensory/motor cortex of rats. In all cases, PV+ neurons were transduced with high efficiency and CR+ neurons were not. We conclude that AAV-mDLX5/6 vectors are biased toward PV+ neurons and away from CR+ neurons.

In the neocortex and hippocampus of rats and primates, PV+ neurons suppress projection neurons (Packer and Yuste, 2011; Hu et al., 2014) whereas some CR+ neurons disinhibit them (Gulyas et al., 1996; Callaway, 2004). These observations, combined with our results, go part of the way toward allaying the concern that stimulation of AAV-mDLX5/6-transduced cells disinhibit projection neurons. However, resolving this issue will require additional experiments, the results of which may depend on species and brain area. Not all CR+ neurons are likely to mediate disinhibition (Barinka and Druga, 2010), disinhibition can be mediated by neurons that are likely CR- (Tremblay et al., 2016), and the complex interconnectivity of excitatory and inhibitory neuronal types undermines intuitions regarding how the activation of one cell type affects others (Litwin-Kumar et al., 2016; Lee et al., 2017).

### AAV tropism

PV+ neurons in the cerebral cortex have larger cell bodies than CR+ neurons, they express different surface proteins, and they have a characteristic laminar density profile (Huang and Paul, 2019; Kooijmans et al., 2020). Any or all of these properties might render PV+ neurons more susceptible to AAV-mediated transduction. However, inherent AAV tropism is unlikely to account for our results. Injections of AAV-hSyn vectors into the neocortex of mice and rats (with AAV9 and AAV1 capsids, respectively) transduced CR+ neurons roughly in proportion to their prevalence (Nathanson et al., 2009; Keaveney et al., 2018). Similar results have been obtained in other parts of the nervous system (Kells et al., 2004; Ivanova et al., 2010; Nieuwenhuis et al., 2023). Anecdotally however, we find that large neurons are transduced more efficiently than small ones. Assessing the impact of this effect on transduction biases across transcriptomic types remains an important research direction.

Laminar biases are also unlikely to account for our results. We observed strong transduction in the supragranular layers of area V1, in which CR+ neurons and AAV-transduced neurons were numerous and intermixed but formed non-overlapping populations (Figure 2A). Moreover, PV+ neurons are densest in layer 4C of V1, a layer that was transduced relatively inefficiently (see Figure 1A and El-Shamayleh et al., 2016).

### Relationship to previous studies

DLX5/6 enhancers similar to the one that we used drive transgene expression in CR+ neurons of transgenic mice, underscoring a difference between germline transgenesis in mice and AAV-mediated gene delivery in other animals (Zerucha et al., 2000; Stühmer et al., 2002; Miyoshi et al., 2010; Wang et al., 2010). One explanation for this difference is that the 862-bp mDLX5/6 enhancer-promoter combination we used (537 bp enhancer) is sufficient to drive expression in some GABAergic neurons, including PV+ neurons, but that additional genetic material is necessary to drive expression in CR+ neurons. Another possibility is that the range of neuronal types in which the DLX5/6 enhancer is active may narrow over the course of development, perhaps being inclusive of CR+ neurons at early stages but not later ones. Finally, this enhancer may be active in different cell types in different species. We note that CR is a more definitive marker for neuronal types in rats and macaques than it is in mice (Gabbott and Bacon, 1997; Xu et al., 2006; Gonchar et al., 2008; Riedemann, 2019).

The selectivity of AAV-mDLX5/6 vectors for PV+ neurons that we observed in this study was lower than in a previous one from our lab (De et al., 2020). This result could be due to differences between the AAV vectors including serotype, payload, the amount of virus injected, or lot-to-lot variation between vectors. Other potential contributing factors include differences in histological methods such as staining protocol, microscopy technique (confocal vs. epifluorescence), and cell counting method (fully manual vs. computer assisted). Co-expression analysis, even with computer assistance, has a subjective component. To minimize this component, nuclear-localized transgene products could be used to increase the signal to noise in cell imaging.

At least three studies besides ours used DLX5/6 enhancers for gene delivery to neurons and looked for biases across GABAergic cell types. None observed the bias that we did. In the first, plasmid DNA containing the GFP gene under the control of the mDLX5/6 enhancer was delivered to the brains of E15.5 mice by *in utero* electroporation (De Marco Garcia and Fishell, 2014). After tissue collection at P8 or P15, most GFP+ neurons expressed NPY, Reelin, VIP, or CR, consistent with a developmental origin in the caudal ganglionic eminence. In contrast, neurons derived from the medial ganglionic eminence (MGE), which includes PV+ neurons, rarely expressed GFP. The selective avoidance of MGE-derived neuronal subtypes in these experiments may have been due to the developmental stage at which the genetic manipulation was made.

The second study was the first to deliver the mDLX5/6 enhancer by intracortical injection of AAV (Dimidschstein et al., 2016). Transgene expression was restricted to GABAergic neurons in all species tested, and, in adult mouse cortex, was detected in PV+, SST+ and VIP+ inhibitory interneurons in approximate proportion to their prevalence. These results are consistent with the idea that AAV-mDLX5/6 vectors are pan-GABAergic. Nevertheless, CR staining was not performed in these experiments, and the possibility remains that CR+ neurons were transduced inefficiently. VIP+ neurons were transduced, and many VIP+ neurons also express CR, but VIP+ and CR+ populations are not identical (Gonchar et al., 2008; Caputi et al., 2009).

The third study applied AAV vectors directly to cultured slices of human medial temporal cortex (Mich et al., 2021). Transduced neurons were collected via fluorescence-activated cell sorting and subjected to RNA sequencing to identify each one’s transcriptomic type. CR expression was not reported, but VIP+ neurons comprised approximately half of the transduced neurons. This study also showed, however, that the neuronal types transduced by AAV vectors in slice culture differed from those transduced *in vivo*. This fact was demonstrated with vectors containing other enhancers, but may extend to those containing the DLX5/6 enhancer.

### Reduction in PV immunoreactivity at injection sites

Cell counting in our study was complicated by a loss of parvalbumin immunoreactivity at some AAV injection sites. One possibility is that transgene overexpression in PV+ neurons reduced PV immunoreactivity. In rat 3 in particular, the immediate injection site was almost devoid of PV+ neurons, so cell counting was restricted to the edges of the transduced area. The fact that other neurons within the same tissue section were clearly PV+ shows that the absence of PV signal was not due to staining reagents or protocol. If neurons that usually express a marker gene fail to do so near some AAV injection sites, selectivity estimates will be biased downward. We attempted to minimize this bias by counting cells in regions where PV, CR, and immunohistochemically amplified transgene were detectable.

### Future directions

Complete characterization of AAV-delivered enhancer activity in adult animals remains an important goal for science and, ultimately, for gene therapy. Major barriers include the lack of appropriately selective antibodies for the identification of cell types post-mortem and the lack of high-throughput *in vivo* reporter gene assays. Straightforward scale-up of conventional reporter gene assays is hobbled by crosstalk between enhancer-reporter pairs, the large number of cells that must be profiled to characterize highly selective enhancers, and the current inability to transduce neurons at brain-wide scale in most animals.

A potentially fruitful way forward is to develop computational models for accurate *in silico* predictions of enhancer activity (Zou et al., 2019). These models have the advantages that they are able to capture complex relationships between genetic sequences and gene expression without the need for manual feature extraction. Disadvantages are that they provide minimal mechanistic insight and require large ground-truth datasets for training. These shortcomings underscore the need for continued basic research in AAV biology, vector-host interactions in the brain, and mechanisms of gene regulation under experiment-relevant conditions. Such data would provide valuable constraints to these models. Discoveries in these areas may guide neuroscientists to the most influential and controllable experimental variables. Reciprocally, discoveries regarding the cell types that are transduced by AAVs carrying *cis-*regulatory elements may shed light on the mechanisms of gene regulation in adult animals.

## Materials and methods

### Ethical approval and animal welfare compliance

All experimental procedures involving animals received approval from the University of Washington Institutional Animal Care and Use Committee and were conducted according to internationally accepted standards. Euthanasia was performed in accordance with the American Veterinary Medical Association (AVMA) guidelines for the Euthanasia of Animals. Four rhesus monkeys (Macaca mulatta) and three Long Evans rats were used in this study (see Table 1).

### Viruses and injections

The mDLX5/6 enhancer was cloned from pAAV-mDlx-ChR2-mCherry-Fishell-3 (Addgene #83898) using In-Fusion assembly (Takara, Inc.). The AAV backbone plasmids that included ChrimsonR-tdTomato and ChRmine-mScarlet were obtained from Addgene (#59171 and #130995, respectively). The rep/cap plasmids for AAV(PHP.eB), AAV1, and AAV9 were generous gifts from Viviana Gradinaru and Benjamin Deverman (Caltech), James Wilson (UPenn), and R. Jude Samulski (UNC Chapel Hill), respectively.

The AAV(PHP.eB)-mDLX5/6-ChrimsonR-tdTomato vector was purchased from the UPenn Vector Core (RRID:SCR_022432). Other AAV vectors were produced in the lab using a conventional three-plasmid transient transfection of human embryonic kidney cells (HEK293T, female, unauthenticated). Vectors were purified by ultracentrifugation through an iodixanol gradient and exchanged into phosphate buffered saline (PBS). The titer of the purified vectors was determined by quantitative polymerase chain reaction.

Monkey A was injected with AAV1-mDLX5/6-ChR2-mCherry through a surgically implanted recording chamber over area V1. Histological data from this animal, not overlapping with those presented here, were published previously (De et al., 2020). Monkey V was injected with AAV9-mDLX5/6-ChR2-mCherry into the prelunate gyrus (presumed area V4). Monkeys J and M were injected with AAV(PHP.eB)-mDLX5/6-ChRmine-mScarlet into the inferior frontal gyrus (presumed frontal eye fields, confirmed electrophysiologically in Monkey M). All three rats were injected with AAV(PHP.eB)-mDLX5/6-ChrimsonR-tdTomato into the somatosensory/motor cortex (approximately 0.5 mm anterior and 2 mm lateral to bregma). Rat 2 was also injected with AAV1-mDLX5/6-ChrimsonR-tdTomato. At the end of each injection, the needle (rat 1) or glass pipette (rats 2 and 3) was left in place for 5–10 min to prevent backflow.

### Tissue preparation

Animals (besides monkey M) were euthanized after a survival period (see Table 1) via sodium pentobarbital overdose and perfused transcardially with 4% paraformaldehyde (wt/vol). Following the perfusion, the brain was removed, post-fixed in 4% paraformaldehyde for 24 h at 4°C, and then transferred to 30% sucrose (wt/vol), and stored at 4°C. Monkey M died unexpectedly. His brain was collected within a few hours, fixed by immersion in 10% formalin, and cryoprotected in 30% sucrose.

### Immunohistochemistry

Tissue blocks were frozen and cut into sections (30–50 μm thick) on a sliding microtome and stored in PBS+ 0.02% sodium azide at 4°C. Free-floating tissue sections were permeabilized and blocked for non-specific antibody binding in blocking solution (PBS with 5% normal donkey serum + 0.2% bovine serum albumin + 0.3% Triton X-100) for 2 h at room temperature (RT) in a 12-well polystyrene plate. All antibodies were diluted in blocking solution, and all incubations were performed with gentle agitation on an orbital shaker. Tissue sections were incubated with primary antibodies for 12–72 h at 4°C (see Table 2 for details on primary and secondary antibodies). Sections from monkeys A and V and rat 1 were stained for the fluorescent protein encoded by the AAV vector (mCherry or tdTomato) and either PV or CR. Sections from monkeys J, M, and rats 2 and 3 were stained for mScarlet or tdTomato (red), PV (green), and CR (far red). After each antibody incubation, free-floating sections were washed five times in PBS. Sections were then incubated in secondary antibodies with DAPI for 2 h at RT. Sections were washed, mounted onto glass slides, and cover slipped using Prolong gold mounting medium (Thermo cat P36930).

**TABLE 2:** Antibodies used for immunohistochemistry.

Antibody target	Host	Source	Product Number	Dilution	RRID
Parvalbumin (PV)	Rabbit	Swant	27	1:5,000	RRID:AB_2631173
Parvalbumin (PV)	Mouse	Swant	235	1:5,000	RRID:AB_10000343
Calretinin (CR)	Rabbit	Swant	7697	1:1,000	RRID:AB_2721226
Red fluorescent Protein (RFP)	Rabbit	Rockland	600-401-379	1:500	RRID:AB_2209751
Red fluorescent Protein (RFP)	Chicken	Rockland	600-901-379	1:500	RRID:AB_10704808
mCherry	Rabbit	Genetex	GTX59788	1:500	RRID:AB_10721869
mCherry	Mouse	Takara	632543	1:500	RRID:AB_2307319
Anti Rabbit AF 488	Donkey	Thermo	A21206	1:500	RRID:AB_2535792
Anti Rabbit AF 568	Donkey	Thermo	A10042	1:500	RRID:AB_2534017
Anti Rabbit AF 647	Donkey	Thermo	A31573	1:500	RRID:AB_2536183
Anti Mouse AF 488	Donkey	Thermo	A21202	1:500	RRID:AB_141607
Anti Mouse AF 568	Donkey	Thermo	A10037	1:500	RRID:AB_2534013
Anti Chicken AF 568	Donkey	Thermo	A78950	1:500	RRID:AB_2921072

### Confocal microscopy

After immunostaining, tissue sections were imaged on a confocal microscope (Leica SP8X) equipped with a tunable laser (470–670 nm) and HyD detectors. Laser intensity and gain were optimized for each channel to minimize background fluorescence and to maximize signal intensity. Z-stack images were obtained using objectives HC PL APO CS 10X/0.40 DRY and HC PL APO CS2 20X/0.75 DRY. The pixel dwell time was 600 ns, and images were acquired at a resolution of 1024 × 1024 pixels.

### Image analysis

Digitized confocal images were analyzed using Fiji (Schindelin et al., 2012) and Imaris software (Oxford Instruments, Zurich, Switzerland). Fiji was used to inspect all images for proper focus and a lack of artifacts. Imaris was used for cell counting. Automated cell counting was achieved using the “spots” function in Imaris with background subtraction. Parameters of the spots function were manually optimized for each marker to minimize false positive and false negative detections, assessed by eye. Images were then manually scanned throughout a superimposed grid to add or remove mislabeled cells. Co-localization analysis was performed using the Matlab-implemented Colocalize Spots plug-in.

## Data availability statement

The original contributions presented in the study are included in the article/supplementary material, further inquiries can be directed to the corresponding author.

## Ethics statement

The animal study was approved by the University of Washington Institutional Animal Care and Use Committee. The study was conducted in accordance with the local legislation and institutional requirements.

## Author contributions

PY-S: Formal analysis, Investigation, Writing—original draft, Writing—review and editing. SG: Formal analysis, Investigation, Writing—original draft, Writing—review and editing. JL: Formal analysis, Writing—original draft. GH: Supervision, Writing—original draft, Writing—review and editing.
